# *CRb* and *PbBa8.1* Synergically Increases Resistant Genes Expression upon Infection of *Plasmodiophora brassicae* in *Brassica napus*

**DOI:** 10.3390/genes11020202

**Published:** 2020-02-17

**Authors:** Nadil Shah, Qian Li, Qiang Xu, Ju Liu, Fan Huang, Zongxiang Zhan, Ping Qin, Xueqing Zhou, Wenlin Yu, Li Zhu, Chunyu Zhang

**Affiliations:** 1 National Key Lab of Crop Genetic Improvement and College of Plant Science and Technology, Huazhong Agricultural University, Wuhan 430070, China; plantpathologist1977@webmail.hzau.edu.cn (N.S.); liqian_hzau@163.com (Q.L.); fhuang@webmail.hzau.edu.cn (F.H.); Qping0187@163.com (P.Q.); xqzhou@webmail.hzau.edu.cn (X.Z.); 13787316246@163.com (W.Y.); 2 Jingmen Agricultural Technology Extension Center, Jingmen 448000, China; xuqiangjm163163@163.com (Q.X.); liujujm163163@163.com (J.L.); 3 Molecular Biology of Vegetable Laboratory, College of Horticulture, Shenyang Agricultural University, Shenyang 110866, China; zhanxiang@syau.edu.cn; 4 Collaborative Innovation Center for the Characteristic Resources Exploitation of Dabie Mountains and the College of Biology and Agricultural Resources, Huanggang Normal University, Huanggang 438000, China

**Keywords:** clubroot, *Plasmodiophora brassicae*, *Brassica napus*, RNA-Seq, disease resistance, molecular mechanisms

## Abstract

*PbBa8.1* and *CRb* are two clubroot-resistant genes that are important for canola breeding in China. Previously, we combined these resistant genes and developed a pyramid-based, homozygous recurrent inbred line (618R), the results of which showed strong resistance to *Plasmodiophora brassicae* field isolates; however, the genetic mechanisms of resistance were unclear. In the present work, we conducted comparative RNA sequencing (RNA-Seq) analysis between 618R and its parental lines (305R and 409R) in order to uncover the transcriptomic response of the superior defense mechanisms of 618R and to determine how these two different resistant genes coordinate with each other. Here, we elucidated that the number and expression of differentially expressed genes (DEGs) in 618R are significantly higher than in the parental lines, and *PbBa8.1* shares more DEGs and plays a dominant role in the pyramided line. The common DEGs among the lines largely exhibit non-additive expression patterns and enrichment in resistance pathways. Among the enriched pathways, plant–pathogen interaction, plant hormone signaling transduction, and secondary metabolites are the key observation. However, the expressions of the salicylic acid (SA) signaling pathway and reactive oxygen species (ROS) appear to be crucial regulatory components in defense response. Our findings provide comprehensive transcriptomic insight into understanding the interactions of resistance gene pyramids in single lines and can facilitate the breeding of improved resistance in *Brassica napus*.

## 1. Introduction

Rapeseed (*Brassica napus*) is the second most important oilseed crop in the world, providing edible oil and raw material for bio-energy applications [1]. China is the second-largest producer of rapeseed, which is the fourth-largest crop in this country after rice, wheat, and maize [2]. It is a typical temperate climate zone oilseed crop cultivated worldwide, especially in the European Union, Canada, and Asia [3].

*Plasmodiophora brassicae* (*P. brassicae*), which causes clubroot disease, is one of the most serious threats to *B. napus* production worldwide, as well as in China. From the first reports, from Taiwan and Fujian in 1912 and 1947, respectively, and later in Jiangxi Province, the pathogen has rapidly spread to other parts of the country, causing severe annual losses of cruciferous crops. It has been estimated that 3.4 to 4 million hectares of farmland are abandoned annually, yielding losses that are estimated to be between 20% and 30% [4,5]. The pathogen has three life-cycle stages, namely, the resting spore stage, root hair infection stage, and cortex infection stage [6]. Primary infection occurs when resting spores (zoospores) germinate and penetrate the host and then grow into multinucleated plasmodia [6]. Gall proliferation on infected roots is the most characteristic symptom obstructing nutrient uptake, resulting in slow growth rates and untimely ripening [7]. The clubroot pathogen is an obligate biotroph and can survive in soil for more than 20 years; thus, it is very difficult to control the disease with chemicals or other mechanical methods once the soil is contaminated [8,9]. Therefore, the development and cultivation of resistant varieties are the most economical and effective approaches to control clubroot disease. 

The pathogen enters the plant through root hairs or wounds, where it then colonizes, multiplies, and injects many immune-suppressing molecules. The plant starts developing symptoms such as gall formation on the roots, followed by yellowing and wilting of the above-ground parts of the plant. 

In order to protect against biotic stresses, plants recognize invading microorganisms via two different recognition systems: (1) pathogen-associated molecular pattern (PAMP)-triggered immunity (PTI) and (2) effector-triggered immunity (ETI), recognized by intracellular receptors [10]. PAMPs are structural molecules that recognize the pathogen at the host surface cell level and induce PTI through pattern recognition receptors (PRR), which trigger a mitogen-activated protein kinase (MAPK) cascade as part of the defense response [11]. The pathogen secretes a series of effectors within the host cell that inhibit recognition by the host, thereby interacting with resistant (R) proteins and initiating the second system of plant immunity, which includes leucine-rich repeats (LRR) or the LRR-like family, the toll/interleukin-1-receptor (TIR), and serine/threonine kinases (S/TK), which trigger a hypersensitive reaction in the plant, inhibiting the spread of the pathogen to adjacent healthy cell tissue and resulting in the plant acquiring systemic resistance to the pathogen [12]. 

RNA-Seq is technique that allows fast and extensive study of transcriptional levels of gene expression. Previously, a number of research articles published transcriptome analysis results of the defense responses of *Brassica* crops against *P. brassicae* at early infection stages at different time points, and resistant and susceptible lines were compared [13,14]. The results of these studies have shown that despite the presence of more robust effector-triggered immunity, the salicylic acid (SA) signaling pathway plays a pivotal role in clubroot resistance in *Brassica rapa*, which contains the *CRb* resistance gene [13], and strongly and rapidly accelerates receptor kinase and G proteins in *B. napus* (ZHE-226) resistance lines that contain the *PbBa8.1* resistance gene, which may trigger reactive oxygen species (ROS), programed cell death (PCD), and the hormonal signal transduction signaling pathway [15]. The current study is different from previously published work. Previous studies were based on lines that were resistant or susceptible to clubroot isolates, and the resistant lines consisted of single resistance genes, in either a homozygous or heterozygous state at the locus. In this study, we pyramid two resistant genes (*CRb* and *PbBa8.1*) and develop a homozygous line. The lines show strong resistance against a number of *P. brassicae* field isolates compared with heterozygous pyramided *B. napus* and single resistant homozygous and heterozygous lines [16]. Historically, the molecular mechanism of the strong resistance of pyramided lines has been poorly understood. Therefore, a comparative transcriptomic analysis between 618R and its parental lines (305R and 409R) is performed in order to understand the superior resistance mechanisms of the pyramided line and to elucidate the genetic compatibility of the two resistant genes in a single line. The current data will unveil the respective resistant pathways for further understanding of the above molecular mechanisms, as well as breeding procedures for *P. brassicae*-resistant canola plants.

## 2. Materials and Methods

### 2.1. Plant Materials

Three *B. napus* clubroot resistant homozygous lines, 305R, 409R, and 618R, carrying the resistant alleles *PbBa8.1*, *CRb*, and *PbBa8.1 + CRb,* were inoculated in a *P. brassicae* suspension. Line 305R, derived from ECD04 and 409R, was derived from the CR Shinki cultivar, whereas 618R, containing two resistant genes (*PbBa8.1* and *CRb*), was developed from the 305R and 409R lines based on marker-assisted selection [16]. A susceptible line was used as control for disease assessment and gall formation analysis. 

### 2.2. Pathogen Isolates

Severely infected clubroot root galls were collected from Zhijiang, Hubei, China, and the pathogen isolate was identified as pathotype 4, as per Williams’ differential system [17]. Resting spores of *P. brassicae* were isolated from fresh galls. The method we used has been described by Xue et al. [18]. Briefly, fresh gall (5 g) was ground with distilled water in order to be homogenized and then filtered through eight layers of cheesecloth. The filtrate suspensions were centrifuged at 4000 rpm for 10 min (min) using a Heraeus Fresco 21 microcentrifuge (Thermo Fisher Scientific, Osterode am Harz, Germany). The supernatant was discarded, and the pellet was resuspended in 5 mL of 50% sucrose and centrifuged at 2000 rpm for 10 min. The supernatant was transferred into a new 50 mL tube and diluted with 30 mL of double-distilled H_2_O (ddH_2_O) and then centrifuged at 4000 rpm for 10 min. The supernatant was discarded, and 5 mL of ddH_2_O was added to the pellet and then centrifuged at 4000 rpm for 10 min in order to get rid of the residual sucrose. Finally, the supernatant was discarded, the pellet was resuspended in 5 mL of ddH_2_O, and the spore suspension was adjusted to a concentration of 5 × 10^6^ spores/mL with ddH_2_O.

#### Sample Preparation, the Inoculation of Resting Spores, and Disease Investigation

305R, 409R, and 618R seeds were sown in a nursery matrix organic medium (Wuhan Xing Yuxing Biology Science Co. Ltd. Wuhan, China). A susceptible line was used as a control for the confirmation of successful inoculation. The medium was kept in the dark for 48 h at 25 °C. Afterwards, 15 seeds per replicate, with three replications, were sown a chamber with a day/night temperature of 25/20 °C, respectively. After one week, the soil was inoculated with resting spores of *P. brassicae* (1 mL of 5 × 10^6^ spores/mL) at the base of each plant. The mock plants were treated with distilled water in the same way. After 20 days of inoculation, the roots of CR lines were sampled for the analysis of DEGs. Three biological replications (each with 10 plants) were used for the sampling process. In order to confirm the resistance status, some resistant lines, along with susceptible ones, were maintained in the greenhouse for the six weeks after inoculation. 

### 2.3. RNA Isolation

Total RNA samples were extracted from the treated and mock lines with Trizol reagent (Invitrogen, Carlsbad, CA, USA) according to the manufacturer’s instructions. RNA quality was determined using a NanoDrop spectrophotometer (Thermo Fisher Scientific Inc., Wilmington, DE, USA) and 2100 Bioanalyzer (Agilent Technologies, Palo Alto, CA, USA).

#### 2.3.1. cDNA Library Construction and RNA-Seq Sequencing

cDNA library preparation and sequencing were performed using a Hifair II First Strand cDNA Synthesis Kit (gDNA digester plus), produced by the Shanghai Yeasen Biotechnology Company (Shanghai, China). mRNA was isolated using oligo(dT) primers. The mRNA was fragmented into short pieces after the addition of fragmentation buffer, and first-strand cDNA was synthesized with random primers. Then, second-strand cDNA was synthesized using the buffer, deoxyribonucleotide triphosphates (dNTPs), RNase H, and DNA polymerase I. The synthetic cDNA was subjected to end-repair by polymerase with Seloxa adapters, and suitable fragments were retrieved via agarose gel electrophoresis. Polymerase chain reaction (PCR) amplification was carried out to enrich the purified fragments. RNA-Seq was carried out using an Illumina HiSeqTM4000 sequencing platform (Illumina, San Diego, CA, USA).

#### 2.3.2. Sequence Data Analysis and Annotation 

We mapped the sequences to the reference database for the *B. napus* genome (https://www.ncbi.nlm.nih.gov/genome/?term=Bra_napus_v2.0) using TopHat [19]. Unigene sequences were compared with the non-redundant protein (nr) database [20], the Kyoto Encyclopedia of Genes and Genomes (KEGG) pathway database [21], and the Gene Ontology (GO) database [22]. GO functional classification and enrichment analysis of all unigenes were performed using the Top GO software package (https://bioconductor.org/packages/release/bioc/html/topGO.html) in order to view the distribution of gene functions. 

#### 2.3.3. Digital Gene Expression Analysis

Gene expression levels were estimated by calculating the read density as RPKM (reads per kilobase transcriptome per million mapped reads) [23]. The correlation (among the three mock and treated lines) of the detected sequences was estimated using Pearson’s correlation coefficient (*p*-value ≤ 0.001) via the DESeq2 software package (http://www.bioconductor.org/packages/release/bioc/html/DESeq2.html). The p-value obtained via the software was calculated using the Benjamini–Hochberg method [24], and the corrected p-values were used to determine the false discovery rate (FDR). Genes were regarded as significantly differentially expressed if the FDR was <0.01 and if the log2-fold change of the RPKM reads were the same as that described by Chen et al. [13]. Differential patterns of gene expression have been represented by Venn diagrams (https://bioinfogp.cnb.csic.es/tools/venny/). The R software (https://rstudio.com) package was used to analyze the different log fold-change values [25].

### 2.4. Quantitative RT-PCR Analysis

In order to validate the RNA-Seq results, quantitative polymerase chain reaction (qPCR) was performed for 11 randomly selected genes (Supplementary Table S1). RNA was extracted from samples of mock and treated roots from the resistant lines using a plant RNA kit (Omega Bio-Tek, Norcross, GA, USA). RNA concentration was calculated using a NanoDrop 2000 spectrophotometer (Qiangen, Hilden, Germany), as per the manufacturer’s instructions. A Platinum^®^ SYBR^®^ Green qPCR Super Mix-UDG (Uracil DNA glycosylase) kit was used to perform the qPCR. The cycling conditions for the reactions were as follows: 95 °C for 4 min, 40 cycles of 95 °C for 20 s, followed by 60 °C for 20 s, and then 72 °C for 30 s. The relative gene expression levels were tested using the method described by Livak et al. [26]. GraphPad Prism5 was used to illustrate the figures and the Pearson’s coefficient correlation between the RNA-Seq and qRT-PCR DEGs. 

## 3. Results

### 3.1. Disease Symptoms Analysis

The pyramided *B. napus* recurrent inbred line (618R) and its parental lines (305R and 409R) were successfully inoculated with pathogen type 4. Inoculation was confirmed by a control susceptible line planted alongside the resistant line under the control condition (Supplementary Figure S1). 

### 3.2. Comparative RNA-Seq Analysis

To analyze the comparative transcriptome level changes in 305R, 409R, and 618R after *P. brassicae* inoculation, Illumina sequencing was conducted via RNA-Seq of the roots of these three lines before and after artificial pathogen inoculation. A total of 807,688,914 reads were generated by 100 bp end sequencing from the six cDNA libraries (Supplementary Table S2). Of these, 24,186,374, 24,468,879, 21,821,768, 23,129,119, 21,324,961, and 19,525,979 average clean reads were generated from the 618R-M (mock), 305R-M, 409R-M, 618R-T (treated), 305R-T, and 409R-T lines (Supplementary Table S2), respectively. The total average percentages of the uniquely mapped, multiple mapped, and total mapped reads were 60.61%, 23.82%, and 84.43%, respectively. The guanine-cytosine percentage (GC%) percentages of the sequence data from the six libraries were all ~47.37%, and the Q20 and Q30 percentages (reads with an average quality score >20 and >30, respectively) were all >90%, indicating that the accuracy and quality of the sequence data were fit for further analysis. 

### 3.3. Comparative Transcriptome Analysis 

**Figure 1:**
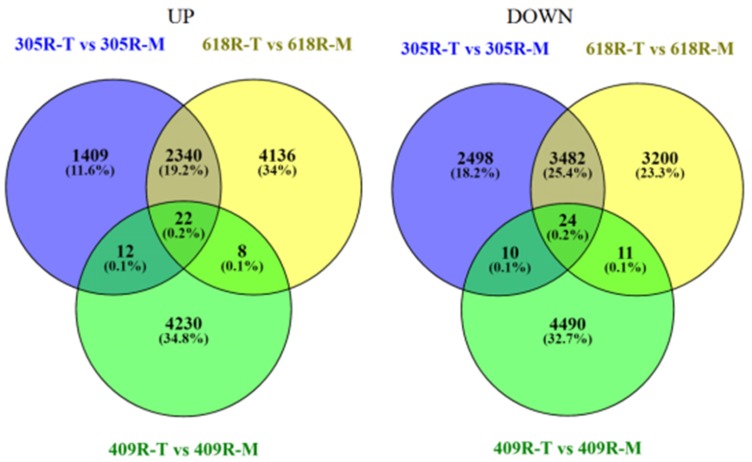
Number of differentially expressed genes that were up- and down-regulated based on a comparison among the resistant lines.

To determine *B. napus* genes that were up-regulated or down-regulated when comparing the mock and treated offspring and parental resistant lines, DeSeq2 was used to yield the log2-fold change value for each gene. For comparison of the lines, a Venn diagram was constructed in order to evaluate the specific up- and down-regulated DEGs. As shown in the Venn diagram (Figure 1), inoculation of the *B. napus* resistant parents and offspring lines with *P. brassicae* resulted in more transcription level changes. A total of 9797, 13,323, and 8807 DEGs were recovered in 305R (305R-T versus 305R-M), 618R (618R-T versus 618R-M), and 409R (409R-T versus 409R-M), respectively. In 305R, 618R, and 409R, 3783, 6506, and 4272 DEGs were up-regulated, respectively, and 6014, 6717, and 4535 DEGs were down-regulated, respectively. Also, 0.2% (22 and 24) of DEGs were identified as common in all three lines that were up-regulated and down-regulated, respectively. Comparatively, 409R shared 0.1% with 618R and 305R, whereas 305R commonly shared 19.2% and 25.4% DEGs with 618R, indicating that *PbBa8.1* is dominant in the considered pyramided lines and shares a greater number of common genes.

### 3.4. Functional Classification of DEGs in P. brassicae Inoculation

**Figure 2:**
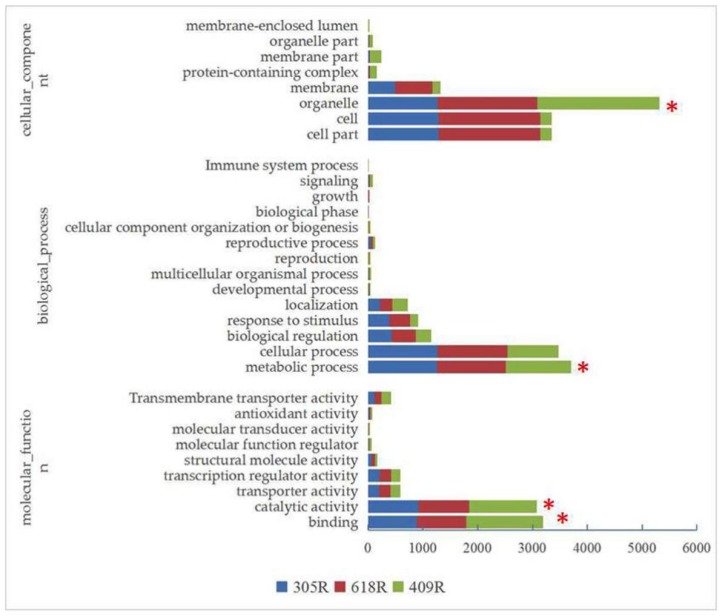
Gene Ontology (GO) enrichment analysis of differentially expressed genes (DEGs) in a comparison among 305R, 618R, and 409R after clubroot pathogen inoculation. “*” indicates the significant differences among three lines.

We carried out GO analysis to clarify the functions of the DEGs in comparison among the three lines after *P. brassicae* inoculation. GO analysis was divided into the cellular component, molecular function, and biological process. For the DEGs, the molecular functions most commonly represented were binding, catalytic activity, and transporter activity, where the cellular component corresponded with cell parts and cell and organelles, whereas the metabolic process, cellular process, and biological regulations and responses to stimulus were the top categories represented by the biological processes in 305R, 618R, and 409R, respectively (Figure 2). Based on the GO analysis, prominent differences in DEGs were detected in the organelles (1273, 1818, 2225), cells and cell parts (1293, 1852, 205), and membrane (499, 671, 152) involved in the cellular components among 305R, 618R, and 409R, while for the molecular function and biological process, differences were observed in binding, catalytic activity, and response to stimulus. This is consistent with DESeq2, although organelles exhibited more DEGs in 409R than in 305R; however, higher numbers of DEGs in 618R were shared by 305R than 409R, indicating that *PbBa8.1* plays a dominant role in the pyramided line. 

**Figure 3:**
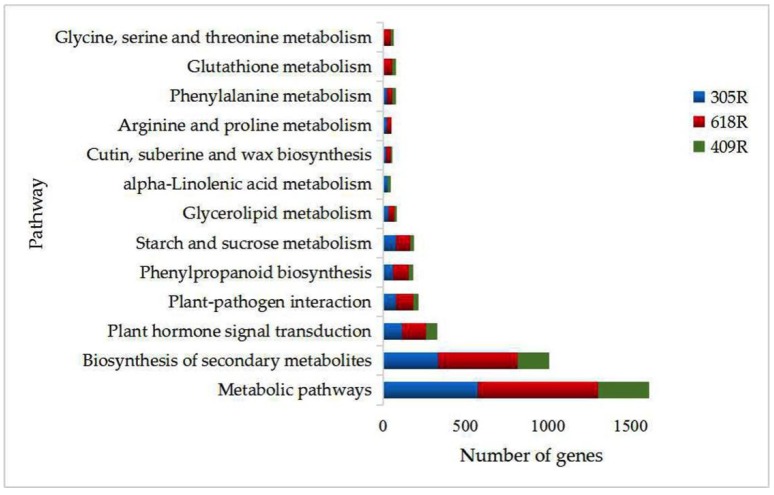
Pathways of 305R, 618R, and 409R enriched in biological processes.

To determine the biological processes that were active in *B. napus*, the differentially expressed unigenes were mapped to the reference KEGG pathways. A total of 2871, 3984, and 1623 DEGs between the treated and mock lines of 305R, 618R, and 409R, respectively, were assigned to 127 KEGG pathways. The most enriched, metabolic pathway in the respective 305R, 618R, and 409R lines contained 573 (19.95%), 728 (18.27%), and 310 (19.10%) DEGs, followed by the biosynthesis of secondary metabolites, i.e., 330 (11.49%), 483 (12.12%), and 191 (11.76%), plant hormone signal transduction, i.e., 115 (4.00%), 145 (3.63%), and 67 (4.12%), and finally, plant–pathogen interaction, i.e., 77 (2.68%), 105 (2.63%), and 32 (1.97%) (Figure 3). Among these processes, plant–pathogen interaction, plant hormone signaling transduction, and phenylpropanoid biosynthesis are normally related to plant disease resistance. The comparative DEG analysis revealed that *PbBa8.1* and *CRb* were constitutively expressed, and the pyramiding of these two genes induced considerably more DEGs compared with either of the single resistant gene lines (Figure 3).

### 3.5. Expressions of DEGs with Different Log-Fold Change Values

The pyramided line was more significant than either of its donor parents with respect to clubroot resistance. Therefore, the gene expression pattern was of significance. The program R was used to determine the different log-fold changes of DEGs between the pyramided lines and their parental lines. Interestingly, the percentage of non-additive expression of DEGs was significantly higher for all log-fold change values (Table 1), and additive expressions were not observed in the pyramided line, which is frequently used to elucidate heterosis. The *PbBa8.1* dominant gene shared a greater number of common genes than *CRb* dominant. The co-dominant gene percentage in the pyramided line was small. However, 10% and 0.3% of DEGs were only expressed in the 305R and 409R lines, respectively, and these were not exhibited in 618R (Table 1).

### 3.6. Identification of DEGs Involved in Resistance to P. brassicae

To determine the defense mechanisms, plant–pathogen interaction, plant hormone signal transduction, phenylpropanoids, and secondary metabolite pathways play important roles in late-stage *B. napus* resistance to *P. brassicae*. To get a deeper insight into the defense mechanisms, overall, 219 resistance-related genes (75.2%, non-additive; 7.9% 305R dominant; 5.9% 409R dominant; 2.0% co-dominant; 7.4% and 1.4% expressed only in 305R and 409R, respectively) were obtained by combining those data in the *B. napus* genome annotation via key word search and with those found from the literature (Supplementary Table S3). The following genes were included: pathogenesis-related (PR) proteins, pattern recognition receptor (PRR) genes, resistance (R) proteins, hormone-regulating genes, calcium influx genes, respiratory burst oxidase homologs (RBOH), mitogen-activated protein kinases (MAPKs), cell wall modification-related genes, salicylic acid (SA), jasmonic acid (JA), ethylene (ET), auxin, and cytokinin genes, as well as WRKY genes. 

#### 3.6.1. Comparative Analysis of the Plant–Pathogen Interaction

The initial step of plant defense against an invading pathogen is triggered by PAMPs, detected by PRRs, leading to PTI. The well-known disease resistance of the nucleotide-binding site leucine-rich repeat (NBS-LRR) protein RPS2 (LOC106362358, LOC106396136, LOC106401114) was abundantly induced in 618R compared to 305R and 409R. One resistance protein (LOC106446267) related to *RMP1* and *RPS3* was only expressed in 618R, and five *R* genes encoding *RML1A* (LOC106376022, LOC106389404, LOC106397401, LOC106451204, LOC106453728) were significantly up-regulated in the pyramided line (Supplementary Table S3). The gene encoding RMP1-interacting protein (LOC106379304), an essential regulator of plant defense, was significantly up-regulated. The genes encoding calcium-dependent protein kinase (*CPK*) were down-regulated, except for *CPK12* and *CPK22*, which were differentially regulated in 618R and 409R as compared to 305R. Similarly, 13, 17, and 8 calcium-binding proteins were down-regulated in 305R, 618R, and 409R, respectively, whereas *CML35* was up-regulated in 618R and *CML19*, which were differentially regulated in 618R and 409R and not expressed in 305R (Supplementary Table S3). The gene encoding *MEKK1* (LOC106367654) was significantly up-regulated in 618R, and down-regulation was observed in 409R. The gene encoding *WRKY33* and six RBOH proteins were down-regulated in the pyramided and parental lines. However, six RBOH proteins were up-regulated in 618R and 305R, while two were down-regulated in 409R. Collectively, these results indicate that 618R has a higher number of NBS-LRR proteins, with higher expressions than the parental lines. However, the RBOH responses were higher in 305R than in 409R, indicating that *PbBa8.1* plays a dominant role over *CRb* in the pyramided line.

#### 3.6.2. Comparative Expression Pattern of Hormone Signaling Transduction

**Figure 4:**
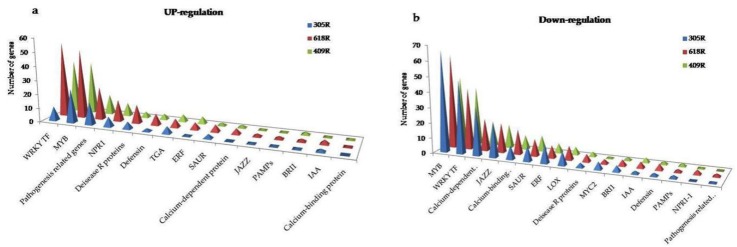
Comparative identification of DEGs related to the plant–pathogen interaction, plant hormonal signal transduction, and transcription factors in the pyramided and parental lines. (**a**) Up-regulation. (**b**) Down-regulation.

Genes associated with hormone signaling were notably affected by *P. brassicae* inoculation. The most significantly affected were ET, JA, SA, abscisic acid (ABA), auxin, and cytokinin, which are all known to play a critical role in plant defense responses and growth development (Figure 4). In the current study, the SA signaling genes were identified as key signaling genes for the clubroot defense response. The genes encoding *NPR1* (LOC106394350, LOC106414379, LOC106431087, LOC106454512) were greatly induced in 618R as compared to 305R and 409R. The genes encoding NIM1-INTERACTING and NIM2-INTERACTING proteins were strongly up-regulated in 618R as compared to 305R and 409R (Supplementary Table S3). Additionally, SUPPRESSOR of *NPR1-1* was greatly down-regulated in 618R as compared to 409R, while it was not expressed in 305R, indicating that the SA signaling pathway is stronger in *CRb* than in *PbBa8.1*. It was found that *TGA7*, *TGA6*, *TGA3*, and *TGA1* were greatly up-regulated in 618R, as compared to *TGA4*, which was differentially regulated in 305R and 618R. In addition, pathogenesis-related (PR1) proteins (LOC106431618) were also induced in 618R. Genes encoding JA biosynthesis and signaling lipoxygenases (*LOX3* and *LOX4*) were significantly reduced in the pyramided and parental lines, while *LOX2* was down-regulated in 305R and 409R and was not expressed in 618R. The genes related to the JA-mediated signaling pathway TIFY (JAZZ) proteins were significantly down-regulated (Figure 4, Supplementary Table S4). *MYC2*, which is a transcriptional activator of ABA and the JA signaling pathway, was greatly down-regulated in 618R and 305R as compared to 409R. In addition, two MAPK-signaling genes (BNAC02G11940D, LOC106389377) related to JA biosynthesis were strongly down-regulated in 618R as compared to 305R and 409R. Two genes related to ethylene receptor 2 (LOC106364668, LOC106446234) were up-regulated in 618R as compared to 305R and 409R, and a gene EIN3-binding F-box (*EFB*) was down-regulated in the pyramided line and 409R, whereas it was not expressed in 305R. The BRASSINOSTEROID INSENSITIVE 1 (*BRI1*) protein, a stress-regulating gene involved in the negative regulation of cell death, was strongly down-regulated in 618R and 305R as compared to 409R. The ABA-INSENSITIVE (ABF) proteins are involved in ABA stress signaling and act as positive components in glucose signal transduction and were significantly down-regulated in the pyramided line and were comparatively differentially regulated in 409R as compared to 305R (Figure 4), indicating that *PbBa8.1* might be profoundly reduced via signaling transduction. Comparatively, an irregularity in expression was found between auxin and cytokinin. The numbers of genes that were almost up- and down-regulated, indicating that these hormones might be involved in plant growth and development, and down-regulated genes may play a key role in the plant defense signaling response activating the SA signaling pathway. Taken together, these results indicate that the pyramided line exhibited a greater amount of plant hormone signaling transduction, with superior expression compared with any of the parental lines.

#### 3.6.3. Comparative Analysis of Pathogenesis-Related (PR) Genes

It is well documented that PR proteins are essential elements of defense proteins concerning pathogen attacks in plants. We identified 26, 19, and 15 PR protein-encoding genes in 618R, 305R, and 409R, respectively (Figure 4, Supplementary Table S4). Here, 13, 7, and 6 PRs were explicitly induced by SA biosynthesis in 618R, 305R, and 409R, respectively. Four PR proteins of the R major gene (LOC106355231, LOC106438995, LOC106440536, LOC106452811), encoding the hypersensitive reaction of pathogens, and 6 *PR1s* (LOC106370793, LOC106431617, LOC106437008, LOC106439741, LOC106444786, LOC106444787) were strongly up-regulated in the pyramided line compared with the parental line.

#### 3.6.4. Comparative Analysis of Transcription Factors (TFs)

It is well known that transcription factors (TFs) play a significant role in plant defense against pathogen invasion. The known TFs *ERF*, *WRKY*, *AUX/IAA*, *MYC2*, and *MYB* have been identified during plant–pathogen interaction (Figure 4). Concerning the genes encoding ET TFs, 5, 1, and 1 genes were up-regulated, while 7, 13, and 6 genes were down-regulated in 618R-T, 305R, and 409R, respectively (Supplementary Table S4). Comparatively, *ERF11* was up-regulated in the pyramided line and 409R, and down-regulation was observed in 305R. *ERF2* was only up-regulated in 409R-T, whereas *ERF11* was up-regulated in 618R-T and 409R-T. The suppression of many WRKY family TF genes was identified. In a comparison of 618R with the parental lines, a number of genes were differentially regulated among the three lines. For example, *WRKY7*, *-56*, *-72*, *-14*, and *-13* were up-regulated in 409R-T, but the suppression of these TFs was recorded in 305R-T and 618R-T. *WRKY48* and *WRKY22* were expressed in 305R-T and 409R-T but not in 618R-T (Supplementary Table S4). *WRKY23* was only highly down-regulated in 305R-T but was up-regulated in 618R-T and 409R-T, while *WRKY40*, *-29*, *-44*, *-33,* and *-6* were down-regulated whereas *WRKY70*, *-38*, *-20*, *-51*, and *-47* were up-regulated. Among them, the *WRKY29* and *WRKY40* TFs were highly suppressed in the pyramided line compared with the parental line. Similarly, an irregularity in the regulation of auxin-responsive proteins and auxin-responsive factors was observed. Auxin-responsive proteins SAUR40 (LOC106387063) and SAUR36 (LOC106443823) were significantly down-regulated, and SAUR50 (LOC106374197) was up-regulated in 618R rather than in 305R and 409R. IAA18 (LOC106346047) was down-regulated, whereas *ARF3* was significantly up-regulated in the pyramided line as compared to the parental lines. *MYC2* was significantly down-regulated in 618R and 305R as compared to 409R. An inconsistency was observed in the *MYB*-related genes (Supplementary Table S4). However, these TFs are also involved in plant growth and development, and the regulation of DEGs may depend upon the response time-periods of the given pathogen and the regulation of plant growth. Together, these results indicate that pyramided *PbBa8.1* and *CRb* elevated gene expression based on plant activity.

#### 3.6.5. Comparative Analysis of Secondary Metabolism

**Figure 5:**
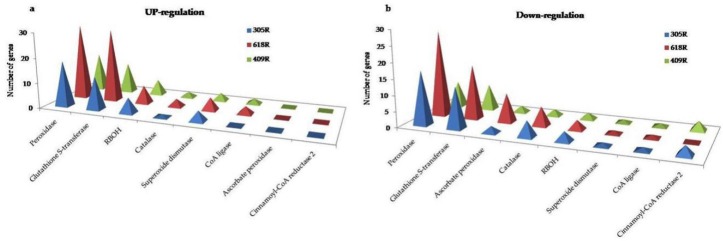
Comparative identification of DEGs related to secondary metabolites, reactive oxygen species (ROS), and antioxidants in pyramided and parental lines. (**a**) Up-regulated genes. (**b**) Down-regulated genes.

Secondary metabolites, such as those produced by the biosynthesis of phenylpropanoids and flavonoids, have been documented to be involved in plant defense responses, such as H_2_O_2_ removal, the oxidation of toxic reductants, lignin degradation and biosynthesis, response to pathogen attack, wounding, and oxidative stress. Genes encoding peroxidases (LOC106417728, LOC106407007, LOC106370453) related to *PER67*, *PER50*, *PER58*, *PRXC2* (LOC106430532), and *HRPN* (LOC106369783) were strongly induced in 618R as compared to 305R and 409R. Interestingly, *PERP7* was only highly expressed in 618R. The gene related to cinnamoyl-CoA reductase (*CCR2*) was down-regulated in the parental lines as compared to 618R. However, the gene related to CoA ligase was differentially regulated in the pyramided and parental lines (Figure 5, Supplementary Table S4). Comparatively, the up-regulation of proteins related to CER1-like 2 was observed in three lines; however, the genes related to peroxygenase were significantly down-regulated, and fatty acyl-CoA reductase and cytochrome were differentially regulated (Supplementary Table S4).

#### 3.6.6. Comparative Analysis of ROS, PCD, and Antioxidant Enzymes

RBOHs are critical enzymes that produce reactive oxygen species (ROS) in response to hormone signaling activated by Ca^2+^, suppressing pathogen growth and regulating host plant cell death (PCD) and the hypersensitive reaction (HR). Seven RBOHs regarded as key factors for the initiation of ROS were significantly up-regulated in 618R, followed by 305R and 409R (Supplementary Table S3), indicating higher ROS activity in the resistant line, inhibiting invasion and the colonization of the clubroot pathogen. A significant expression in gene profiling was observed among genes encoding antioxidant enzymes, such as glutathione S-transferase (GST), superoxide dismutase (SOD), ascorbate peroxidase (APX), and catalase (CAT). Here, 29 GST genes were up-regulated in 618R as compared to 13 in 305R and 8 in 409R (Figure 5). Comparatively, GSTs *US25* and *US5* were differentially regulated in 409R. The antioxidant enzyme that encodes SOD, which leads to an increase in SOD activity when it is increased, was observed to increase in 618R and 305R as compared to 409R. APX maintains ROS homeostasis in plant cells and restricts ROS-dependent signal transduction. *APX1* and *APX2* were down-regulated in 305R-T, 618R-T, and 409R-T. Additionally, in 618R-T, *APX3* and two other putative APXs (*APX6*) were significantly down-regulated. The catalase (*CAT*)-related gene that protects cells from ROS, *CAT3* (LOC106440736), was profoundly expressed in 618R, while *CAT2*-related genes were down-regulated in *PbBa8.1* and *CRb* (Supplementary Table S4). The GST, SOD, APX, and CAT antioxidant ability was higher in 305R than in 409R, indicating that *PbBa8.1* plays a stronger role than *CRb* and that gene expression profiling was elevated in 618R in the presence of these two resistant genes.

#### 3.6.7. Identification of DEGs Involved in Cell Wall Modification

To elucidate the cell wall-related DEGs, we identified arabinogalactan proteins (AGP), cell division (*CDC*), expansin (*EXPA*), pectin methylesterase (*CGR*), and xyloglucan endotransglucosylase (XTH) regulations among the lines. Comparatively, *AGP12*, *AGP2*, and *AGP3* were highly suppressed in all three lines in terms of their number of loci, whereas *AGP16* and *AGP25* were up-regulated. Among the DEGs, *AGP22* was down-regulated in 618R-T and 409R-T and was not expressed in 305R-T. *AGP14* was positively expressed in 305R-T and 618R-T and negatively regulated in 409R-T. CDC proteins were down-regulated in 305R-T and 618R, while LOC106382582 was differentially expressed in 409R-T (Supplementary Table S4). The DEGs encoding *EXPA* were mostly up-regulated when compared with the mocks; however, some DEGs were differentially regulated when compared with the pyramided line. For example, *EXPA A6* and *A13* were up-regulated in 618R-T, down-regulated in 409R-T, and not expressed in 305R-T. The cell wall modification-related DEGs (*CGRs*) were highly suppressed, whereas the *XTH* genes were mostly down-regulated. Collectively, these results indicate that cell wall modification-related genes might not play any significant role in defense mechanisms in this regard.

### 3.7. qRT-PCR Validation

**Figure 6:**
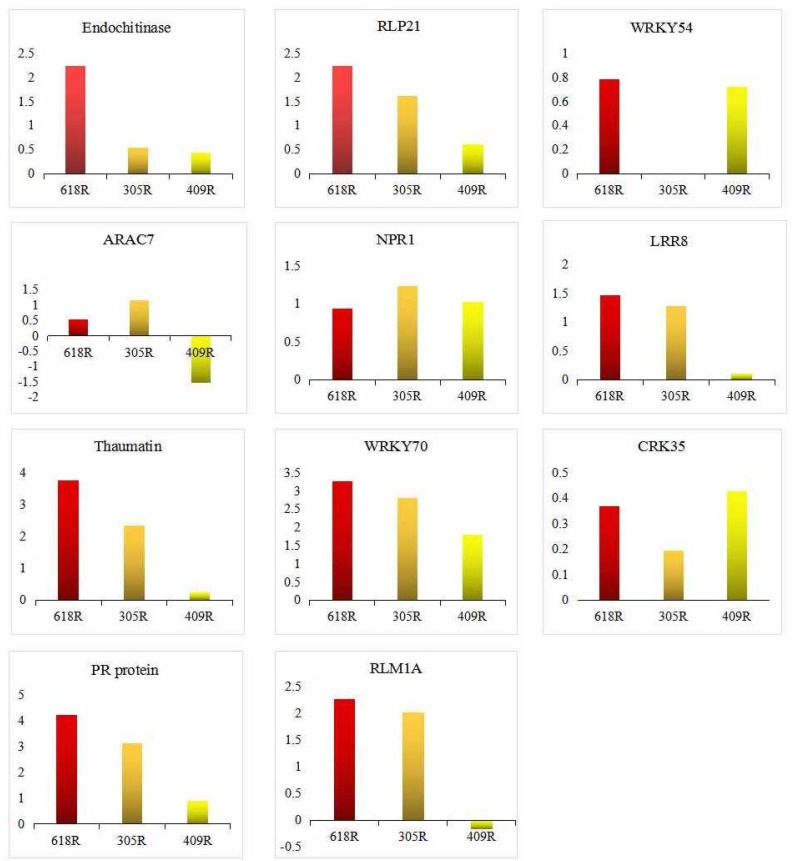
Validation of RNA sequencing (RNA-Seq) data by quantitative real time polymerase chain reaction qRT-PCR.

To validate the RNA-Seq results, 11 genes were randomly selected for qRT-PCR analysis. These genes included a pathogenesis-related protein (thaumatin), a disease resistance protein, endochitinase, a receptor-like protein, an ethylene-responsive transcription factor, WRKY, cysteine-rich receptor-like protein kinase, the NIM1-INTERACTING protein, and a GTP binding protein. For all 11 genes, similar expression tendencies were detected as in the RNA-Seq analyses (Figure 6). The Pearson coefficient correlation (R^2^) levels of 618R and 409R exceeded 0.60, whereas 305R had a coefficient of 0.50 (Supplementary Figure S2). Thus, the results indicate the reliability of our transcriptome analysis data.

## 4. Discussion

The pyramiding of *PbBa8.1* and *CRb* resistant genes strongly induces resistance against a number of *P. brassicae* field isolates [16]. To understand the molecular mechanism of how these two resistance genes elevate resistance in a single line, we selected only one time point to comparatively investigate the genetic coordination of the pyramided line (618R) with its parental lines (305R and 409R), rather than focus on the change in the transcriptome at different time points after clubroot pathogen inoculation. In this study, it was shown that *PbBa8.1* shares a greater number of DEGs than *CRb* and plays a dominant role, with a higher number of non-additive genes than the pyramided line. This insight revealed that the pyramided line strongly triggers multiple resistance pathways, including the plant–pathogen interaction, plant hormone signal transduction, secondary metabolites, transcription factors, and other processes, indicating that the plant has responded via a multi-gene network to pathogen attack. Most interestingly, it was shown that the SA signaling pathway was higher in 409R than 305R, and the ROS response was greater than signaling in 305R. However, higher expressions were evaluated in 618R, indicating that the resistance to *P. brassicae* was mainly controlled by SA and ROS in the pyramided line. These results will provide comprehensive transcriptomic insight into uncovering the interaction of pyramiding resistance genes in a single line, and this architecture further facilitates breeding programs with improved resistance efficiency in terms of *B. napus*.

### 4.1. Schematic Model of the Interaction of the Clubroot Pathogen and B. napus

**Figure 7:**
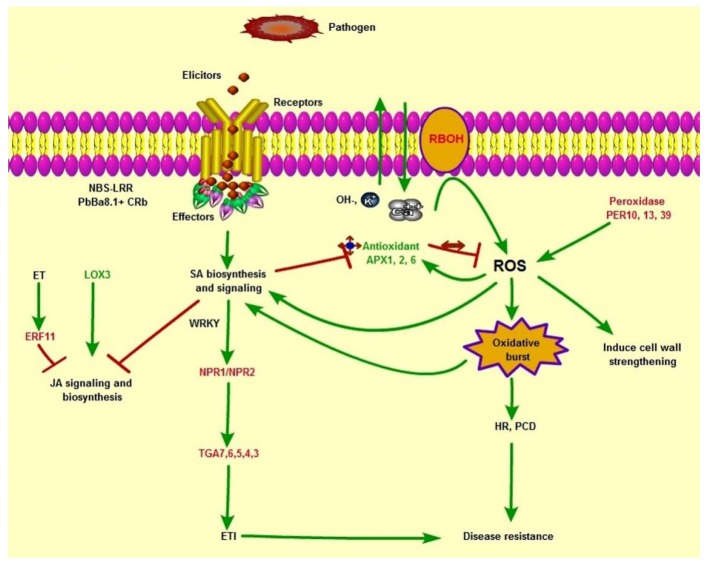
Schematic diagram showing the plant defense signaling network in *B. napus* resistance lines in response to *P. brassicae*.

Based on the comparative transcriptome analysis results, the superior molecular resistance to clubroot is reviewed in Figure 7. In this model, the NBS-LRR protein of PbBa.1 and CRb recognizes the elicitors released by *P. brassicae* and triggers defense signal transduction. The key pathways associated here are SA, JA, MAPK, ET, Ca^2+^, and ROS. The activation of SA signaling transduction and down-regulation via JA signaling and biosynthesis affects the defense-related WRKY and ERF transcription factor families, which regulate downstream defense mechanisms. On the other hand, RBOH and phenylpropanoid biosynthesis activate ROS activities that also enhance the SA signaling and biosynthesis, PCD, and the HR pathway. Antioxidant enzymatic regulations may also contribute to the defense response.

#### 4.1.1. Change in Gene Expressions in Response to *P. brassicae* Inoculation

To cope with *P. brassicae*, a total of 9797, 13,323, and 8807 DEGs were identified in 305R, 618R, and 409R, respectively. qRT-PCR results validated the DEGs selected from transcriptome profiling. The pyramided line, 618R, showed a higher number of DEGs than the parental lines, indicating that 618R may trigger different genes or gene complexes that control the resistance response. However, *PbBa8.1* shared a higher number of DEGs than *CRb*, indicating that it plays a dominant role in the pyramided line. 

We exposed the pyramided and parental *B. napus* lines to the clubroot pathogen *P. brassicae* pathotype 4. Significant changes in plant–pathogen interaction, cell wall modification, plant hormone signaling, metabolic process, and oxidative and plant defense response were observed. 

#### 4.1.2. Plant–Pathogen Interaction

Plants have evolved a series of defense mechanisms in the presence of pathogens during their co-evolution; these mechanisms are instigated by pathogen effectors and host R proteins. PTI is the first defense mechanism, in which plants detect the pathogen through PAMPs regulated by PRR proteins [27]. PTI is involved in the induction of plant hormone signal transduction and plant–pathogen interaction at the late stage of *B. rapa* resistant lines containing the *CRd* gene when exposed to *P. brassicae* [28]. In contrast, in the *B. rapa* near-isogenic line (CR BJN3-2) and *B. napus* resistance line (ZHE-226), no significant changes were detected when compared with the susceptible lines after inoculation with *P. brassicae* [13,15]. In this study, in comparison, we could not detect a significant interaction between the host and pathogen in the first defense mechanism, which might be due to pathogen effector proteins which have evolved to suppress the PTI; however, further study is required. The second layer of immunity is the recognition of effectors, which leads to the initiation of ETI. Plants have evolved specific resistance proteins (R proteins) that recognize pathogenic virulence molecule effectors, leading to ETI [10]. In the current study, we identified 13 NBS-LRR R proteins in 618R, which was two-fold higher than in any of the parent lines (Supplementary Table S4). *RPS2*, *RPS3*, and *RPS5* were strongly induced in 618R as compared to 305R and 409R. It was reported that *RPS2* confers resistance against downy mildew in grapevines [29]. *RPM1* (*RPS3*) and *RPS2* also confer resistance against *Fusarium* wilt in bananas [30]. The strong up-regulation of *R* genes in 618R suggests that the pyramiding of resistance genes enhances the ETI response, which plays a significant role in maintaining host resistance.

Calcium is considered to be an imperative secondary messenger in the signal transduction pathway, which plays a vital role in plants in the production of HR in response to biotic stress [31,32]. In this study, the down-regulation of *CMLs*, *CIPKs*, and *CDPKs* was highly suppressed in 618R as compared to 305R and 409R. These results are similar to those previously described concerning the interaction between *B. rapa* and *P. brassicae*, where up-regulation was seen in CR-BJN-3 at 0 h after inoculation [13]. Ca^2+^ also triggers RBOH proteins, which participate in ROS production. A significant up-regulation of RBOH factors was found in 618R, followed by 305R, indicating higher ROS activity in the pyramided and 305R lines, inhibiting the invasion and colonization of the clubroot pathogen.

#### 4.1.3. Defense Signaling Transduction

Signaling transduction was activated soon after recognition of the pathogen for the establishment of the defense system. The phytohormones SA, JA, and ET are mainly involved in defense processes [30]. *P. brassicae* is a biotrophic pathogen, and resistance is mainly controlled by the SA-mediated signaling pathway in this regard [33]. Our data revealed that SA was significantly accumulated in the pyramided and parental lines, reflecting its crucial role in clubroot defense. This result is in agreement with previous works, which showed that the SA pathway mediates clubroot resistance in *Brassica* crops [13]. The PR protein had a superior impact in the response to *P. brassicae,* serving as a guard to the plants for further infection, the hypersensitive response (HR), or systemic-acquired resistance (SAR) [34]. In our study, we identified a significant induction of PR proteins, thaumatin, and lipid transfer proteins in the pyramided line as compared to the parental lines. Additionally, the induction of defensin (*PDF*) after inoculation with the clubroot pathogen supplemented the PR role in the plant disease resistance response. These findings support the reports showing the up-regulation of PR proteins and *PDF* in plant defense in response to *P. brassicae* in *Brassica oleracea* and *Arabidopsis thaliana* resistant lines [33,34,35]. We concluded that PR proteins are involved in the resistance response against the clubroot pathogen and that the pyramiding of resistance genes enhances PR activities. However, SA is a pivotal factor in the SAR response, and the SA signaling pathway is mainly effective for biotrophic pathogens. NONEXPRESSOR OF PATHOGENESIS-RELATED (NPR) is a master regulator of plant SAR, conferring immunity, and SAR induction leads to the accumulation of SA signaling molecules, resulting in the induction of defense genes through NPR1 activation [36]. NPR1 further interacts with TGA, and these TGA factors bind with *PR1* gene promoters, which is necessary for the SA response [36,37]. In the current study, we found that *NIM1*, *NIM2*, and *PR1* were significantly up-regulated in 618R, followed by 409R and 305R. The suppression of the SA-mediated pathway in susceptible *A. thaliana* and the up-regulation of NPR1 and PR1 genes in CR-BJN3-2 were both reported earlier after inoculation with *P. brassicae* [13,38]. The up-regulation of SA signaling transduction was reported in resistant genotypes [39]. Taken together, these works suggest that enhanced SA levels in host cells suppress *P. brassicae* colonization. 

The interaction between SA and JA is antagonistic [40]. We observed that genes related to JA signaling and biosynthesis (*LOX3*, *LOX4*) were extremely down-regulated in the pyramided and parental lines. However, differential regulations of *LOX2* were detected between 618R, 409R, and 305R. The TIFY protein and *MYC2* are related to JA signaling and are activators, which were significantly down-regulated in the pyramided line as compared to the parental lines. Besides this, three genes of MAPK related to JA biosynthesis were profoundly down-regulated in the pyramided line as compared to the parental lines. This is in agreement with the claim that JA biosynthesis is expressed in disease resistance to necrotrophic pathogens and is suppressed in biotrophic pathogens [30,41]. The stress-regulating gene involved in the negative regulation of cell death, the BRI1 protein, was strongly down-regulated in the pyramided and 305R lines as compared 409R, indicating that *PbBa8.1* plays a dominant role in the pyramided line in this regard. 

ROS is a well-known secondary messenger in cellular processes, and RBOHs are critical enzymes that produce ROS in response to hormonal and environmental signals and are activated by Ca^2+^ [42,43]. ROS-associated signaling that suppresses pathogen growth determines PCD and HR [15]. It also works with other signaling molecules and induces SA and its regulators, the NPR1 and TGA transcription factors [44], and ROS-mediated defense plays a central key role in cross-talk with other molecular mechanisms, having great potential for resistant genotype selection [45]. We observed that six genes related to RBOH, regarded as key initiators of ROS, were strongly induced in 618R and 305R as compared to 409R, indicating a higher ROS ability in *PbBa8.1* than in *CRb*. An elevation of RBOH in resistant lines is in agreement with previous studies in *B. napus* and *B. oleraceae* [14,46]. PERs maintain ROS homeostasis in plant cells and restrict ROS-dependent damage or finely tune ROS-dependent signal transduction with regard to down-regulated scavenging systems [47]. Five genes related to *PER* were strongly down-regulated in 618R as compared with the parental lines; however, *PERP7* was only repressed in 618R. The antioxidant enzymes GST, SOD, APX, and CAT, which play a significant role in the scavenging of ROS, were strongly regulated after the inoculation of *P. brassicae*. SOD activity was greatly induced in 618R and 305R as compared to 409R. APX, which maintains ROS homeostasis in plant cells and restricts ROS-dependent signaling transduction, was down-regulated in the pyramided and 305R lines, while CAT3, which protects cells from ROS, was profoundly expressed in 618R. However, higher ROS activity in the resistance NIL line CR-BJN3-2 [13] was observed, and we concluded that *PbBa8.1* played a stronger role than *CRb*.

#### 4.1.4. Transcription factor (TF) Response to *P. brassicae*

TFs respond soon after the pathogen attack in defense signaling transduction. In this study, *ERF*, *WRKY*, *AUX/IAA*, *MYC2*, and *MYB* were identified during the plant–pathogen interaction. ERF TFs bind to a GCC-motif in the promotor of JA and ET responsive genes, thereby positively or negatively regulating gene expression [48]. Elevation of *ERF11* was observed in 618R-T and 409R-T, and negative regulation was ascertained in 305R-T, indicating that there may be negative regulators in resistance lines containing *PbBa8.1* for the purpose of the down-regulation of the JA signaling pathway. Negative regulations of *ERF11* JA-responsive defense expressions were reported in *B. napus* when exposed to *Fusarium oxysporum* [49]. WRKY TFs bind with downstream elements and play an important role in biotic and abiotic responses [50,51], whereas the *WRKY70* TF represents an antagonistic interaction between SA and JA [52]. It positively regulates the SA-mediated signaling pathway and negatively regulates the JA-mediated pathway, and together with *WRKY TF-54*, *-53*, and *-46*, it promotes the defense response in biotrophs by inducing SA-mediated PR gene expression [52,53]. Interestingly, in our study, the *WRKY70* TF was greatly induced in 618R-T and 409R-T as compared to 305R-T after inoculation with *P. brassicae*, indicating the induction of SA signaling and the repression of JA signaling genes induced by PR gene expression, also enhancing resistance in *B. napus* lines. An inconsistency was observed for auxin-related TFs, such as *SAUR* genes, where down-regulation was observed in *IAA18*. *MYC2* genes were strongly down-regulated in the pyramided and 305R lines as compared to 409R, and related genes were also inconsistent in their regulations. However, these factors are also involved in plant growth and development, and the regulation of these genes might be dependent on the pathogenic response over different time periods. Overall, these results indicate that TFs play an important role in plant defense and that the pyramiding of *PbBa8.1* and *CRb* enhances both the expressions of genes and their number.

## 5. Conclusions

In this study, we carried out comparative transcriptomic analysis of the roots of a recurrent inbred pyramided line, ‘618R’, and its parental lines, 305R and 409R, of *B. napus*, containing the *PbBa8.1* and *CRb* resistance genes. The differences in the number and expressions in terms of DEGs among the lines facilitated a comprehensive overview of the transcriptome profiles in the *P. brassicae* response. We showed that *PbBa8.1* shares a greater number of DEGs than *CRb* and plays a dominant role in the pyramided line. Comparative correlation analysis of different log-fold changes demonstrated a higher number of non-additive genes in the pyramided line. This insight revealed that the pyramided line strongly triggers multiple resistance pathways, including the plant–pathogen interaction, plant hormone signal transduction, secondary metabolites, transcription factors, and other processes, indicating that the plant responds via a multi-gene network to pathogen attack. Comparative analysis revealed that, among the DEGs, some DEGs were differentially regulated and expressed in the pyramided line, perhaps due to cross-talk or over-expression. In addition, we showed that the SA signaling pathway was stronger in 409R, and ROS response was greater than signaling in 305R. However, higher expressions were evaluated in 618R, indicating that the resistance to *P. brassicae* was mainly controlled by SA and ROS in the pyramided line. These results provide a comprehensive transcriptomic insight into uncovering the interaction of the pyramiding of resistance genes in a single line, and this architecture further facilitates breeding programs with the aim of improved resistance efficiency in *B. napus*.

## Figures and Tables

**Table 1:** Comparative gene expression analysis of 618R with 305R and 409R in response to different log change values after *P. brassicae* inoculation.

Log Value	Non-Additive %	Co-Dominant %	*PbBa8.1* Dominant %	*PbBa8.1* Only %	*CRb* Dominant %	*CRb* Only %
<0.1	81.0	0.9	4.2	10.0	3.2	0.3
<0.25	71.1	2.0	8.4	10.1	7.5	0.3
<0.50	61.3	3.4	13.9	10.1	10.6	0.3
<0.75	51.5	3.7	18.2	10.1	15.9	0.3
<1.0	83.6	2.5	5.2	-	2.5	-
<1.25	64.8	3.0	14.5	-	17.5	-

## Supplementary Materials

The following are available online at https://www.mdpi.com/2073-4425/11/2/202/s1, Figure S1: Phenotype of roots in *B. napus* after inoculation of *P. brassicae*; Figure S2: Pearson coefficient correlation of RNA-Seq and qRT-PCR of DEGs. *Y*-axis shows the fold change between mock and treated, whereas the *x*-axis exhibits fold change by qRT-PCR. Table S1: Primer sequence for qRT-PCR; Table S2: Statistics of the sequenced reads and comparison to the *B. napus* reference genome; Table S3: List of DEGs in several metabolism pathways and biological processes involved in resistance for 305R, 618R, 409R *B. napus* resistance lines after inoculation of P. brassicae; Table S4: List of DEGs of 618R, 305R and 409R.
