# Supplementary material for: CRb and PbBa8.1 Synergically Increases Resistant Genes Expression upon Infection of Plasmodiophora brassicae in Brassica napus
Source: Genes (Basel). 2020 Feb 17;11(2):202. doi: 10.3390/genes11020202 (PMC7074261; doi:10.3390/genes11020202)
Supplement: Supplementary file 1 [file genes-11-00202-s001.zip › Supplementary data/Supplementary figures.doc]

Supplementary Fig S1 Phenotype of roots in *B. napus* after inoculation of *P. brassicae*

**Supplementary Fig S2** Pearson coefficient correlation of RNA-Seq and qRT-PCR of DEGs. Y-axis showed the fold change between mock and treated, whereas the x-axis exhibited fold change by qRT-PCR.
