# Supplementary material for: CRb and PbBa8.1 Synergically Increases Resistant Genes Expression upon Infection of Plasmodiophora brassicae in Brassica napus
Source: Genes (Basel). 2020 Feb 17;11(2):202. doi: 10.3390/genes11020202 (PMC7074261; doi:10.3390/genes11020202)
Supplement: Supplementary file 1 [file genes-11-00202-s001.zip › Supplementary data/Supplementary Table S1.doc]

Supplementary Table S1: Primer sequence for qRT-PCR

| **gene_ID** | **Sense** | **Sequence** | **GC%** | **Tm** | **length** |
| --- | --- | --- | --- | --- | --- |
| LOC106355231 | F | CCACCCAGGCTCGGATTT | 61.1 | 60.3 | 114 |
|  | R | CTCGTCCATAAGATTTGCACTCA | 43.5 | 59.8 |  |
| LOC106440536 | F | CGACTCAATCAAGGCGAAAC | 50 | 58.2 | 119 |
|  | R | CCAGTTTGGCATCCACCTC | 57.9 | 58.2 |  |
| LOC106439756 | F | TGATGCTGATGGCGATGGT | 52.6 | 60 | 128 |
|  | R | CTTCCATTTCCTCCGTAGACAGT | 47.8 | 59.5 |  |
| LOC106395467 | F | AAGCGGTGGTGTAGGTCTCA | 55 | 57.8 | 134 |
|  | R | AAGCCTGACGAGTGTAGAAACC | 50 | 58 |  |
| LOC106439065 | F | TCCCGAATACAACTGATAAGGC | 45.5 | 58.8 | 146 |
|  | R | AGAGCCTAAGAAGTCGTTGGAGT | 47.8 | 58.7 |  |
| LOC106397797 | F | CATTTCCCTAATCCTACACCACAA | 41.7 | 60.3 | 83 |
|  | R | CTATCGCCGCTGCTATCCA | 57.9 | 59.7 |  |
| LOC106422388 | F | TCTTCAACTCCTCCATCGGTC | 52.4 | 58.9 | 130 |
|  | R | TAGGACAGCCCTCTAACAATCTCT | 45.8 | 58.8 |  |
| LOC106370082 | F | AGGAAAGAGGTCACTGGGAATAA | 43.5 | 59.1 | 122 |
|  | R | GCTGGGACTTGTCACTTCACTT | 50 | 58.4 |  |
| LOC106374298 | F | GGAGGAAAGAGGTCACTGGG | 60 | 58.2 | 124 |
|  | R | TGCTGGGACTTGTCACTTCACT | 50 | 59.6 |  |
| LOC106445333 | F | GCAAGGCAACAAAGCAAGTA | 45 | 56.8 | 98 |
|  | R | GGTGTTACTTCGTCCGTGTCG | 57.1 | 60.3 |  |
| LOC111205892 | F | ACCCGTATTGTTTCTGGCGAT | 47.6 | 61.1 | 108 |
|  | R | CATAGGGACGGAGTCACAAGAA | 50 | 59.3 |  |
