# Supplementary material for: CRb and PbBa8.1 Synergically Increases Resistant Genes Expression upon Infection of Plasmodiophora brassicae in Brassica napus
Source: Genes (Basel). 2020 Feb 17;11(2):202. doi: 10.3390/genes11020202 (PMC7074261; doi:10.3390/genes11020202)
Supplement: Supplementary file 1 [file genes-11-00202-s001.zip › Supplementary data/Supplementary Table S2.doc]

**Supplementary Table S2.** Statistics of the sequenced reads and comparison to the *B. napus* reference genome

| **Sample ID** | | **Read Number** | **Total Bases** | **Raw GC content(%)** | **Raw Q20 rate(%)** | **Raw Q30 rate(%)** | **Total Clean reads** | **Uniquely mapped(%)** | **Multi mapped(%)** | **Total mapped (%)** |
| --- | --- | --- | --- | --- | --- | --- | --- | --- | --- | --- |
| **618R- Mock** | A1 | 52139236 | 7841405568 | 47.29 | 97.98 | 95.02 | 26069618 | 55.47 | 28.56 | 84.03 |
|  | A2 | 45575552 | 6852908344 | 47.27 | 98.07 | 95.19 | 22787776 | 53.98 | 28.52 | 82.5 |
|  | A3 | 47403458 | 7126508996 | 47.37 | 98.1 | 95.26 | 23701729 | 54.07 | 28.47 | 82.54 |
|  | **Average** | **48372748.67** | **7273607636** | **47.31** | **98.05** | **95.15** | **24186374.33** | **54.50** | **28.51** | **83.023** |
| **305R- Mock** | B1 | 52292692 | 7860594390 | 47.14 | 98.17 | 95.42 | 26146346 | 54.56 | 27.87 | 82.43 |
|  | B2 | 47911514 | 7204332400 | 47.21 | 98.01 | 95.12 | 23955757 | 55.89 | 27.78 | 83.67 |
|  | B3 | 46609072 | 7006515342 | 47.38 | 98.09 | 95.23 | 23304536 | 55.3 | 28.03 | 83.33 |
| **Average** | | **48937759.33** | **7357147377** | **47.24** | **98.09** | **95.25** | **24468879.67** | **55.25** | **27.89** | **83.1** |
| **409R-mock** | C1 | 41989642 | 6764573491 | 47.28 | 98.78 | 96.35 | 20918305 | 73.84 | 15.9 | 89.74 |
|  | C2 | 47087236 | 7580617277 | 47.24 | 98.78 | 96.4 | 23453033 | 73.75 | 15.72 | 89.47 |
|  | C3 | 42349128 | 6828998001 | 47.27 | 98.74 | 96.28 | 21093967 | 73.95 | 15.54 | 89.49 |
| **Average** | | **43808668.67** | **7058062923** | **47.26** | **98.76** | **96.34** | **21821768.33** | **73.84** | **15.72** | **89.56** |
| **618R- Treated** | D1 | 44786132 | 6731157682 | 47.59 | 98.09 | 95.26 | 22393066 | 52.28 | 27.24 | 79.52 |
|  | D2 | 49281094 | 7409661524 | 47.6 | 98.15 | 95.33 | 24640547 | 52.96 | 27.69 | 80.65 |
|  | D3 | 44707490 | 6722046010 | 47.56 | 98.07 | 95.18 | 22353745 | 56.61 | 27.18 | 83.79 |
| **Average** | | **46258238.67** | **6954288405** | **47.58** | **98.10** | **95.25** | **23129119.33** | **53.95** | **27.37** | **81.32** |
| **305R- Treated** | E1 | 39690012 | 5966113870 | 47.61 | 97.52 | 93.68 | 19845006 | 53.16 | 27.65 | 80.81 |
|  | E2 | 47455254 | 7132533218 | 47.5 | 97.59 | 93.89 | 23727627 | 53.28 | 27.85 | 81.13 |
|  | E3 | 40804500 | 6133395340 | 47.61 | 97.5 | 93.62 | 20402250 | 52.27 | 27.87 | 80.14 |
| **Average** | | **42649922** | **6410680809** | **47.57** | **97.53** | **93.73** | **21324961** | **52.90** | **27.79** | **80.69** |
| **409R-Treated** | F1 | 37845130 | 6098853560 | 47.21 | 98.72 | 96.24 | 18849474 | 73.2 | 15.72 | 88.92 |
|  | F2 | 40847676 | 6571299963 | 47.33 | 98.76 | 96.36 | 20345669 | 73.35 | 15.51 | 88.86 |
|  | F3 | 38914096 | 6270652252 | 47.22 | 98.76 | 96.35 | 19382796 | 73.14 | 15.64 | 88.78 |
| **Average** | | **39202300.67** | **6313601925** | **47.25** | **98.74** | **96.31** | **19525979.67** | **73.23** | **15.6** | **88.85** |
| **Total Average** | | **807688914** | **1.24102E+11** | **47.37** | **98.21** | **95.343** | **403371247** | **60.61** | **23.81** | **84.43** |
|  |  |  |  |  |  |  |  |  |  |  |
